# Immobilization of an amino acid racemase for application in crystallization‐based chiral resolutions of asparagine monohydrate

**DOI:** 10.1002/elsc.202000029

**Published:** 2020-08-28

**Authors:** Thiane Carneiro, Katarzyna Wrzosek, Katja Bettenbrock, Heike Lorenz, Andreas Seidel‐Morgenstern

**Affiliations:** ^1^ Max Planck Institute for Dynamics of Complex Technical Systems Magdeburg Germany; ^2^ Otto‐von‐Guericke University Magdeburg Magdeburg Germany

**Keywords:** amino acid racemase, enzyme immobilization, packed bed reactor, racemization

## Abstract

Integration of racemization and a resolution process is an attractive way to overcome yield limitations in the production of pure chiral molecules. Preferential crystallization and other crystallization‐based techniques usually produce low enantiomeric excess in solution, which is a constraint for coupling with racemization. We developed an enzymatic fixed bed reactor that can potentially overcome these unfavorable conditions and improve the overall yield of preferential crystallization. Enzyme immobilization strategies were investigated on covalent‐binding supports. The amino acid racemase immobilized in Purolite ECR 8309F with a load of 35 mg‐enzyme/g‐support showed highest specific activity (approx. 500 U/g‐support) and no loss in activity in reusability tests. Effects of substrate inhibition observed for the free enzyme were overcome after immobilization. A packed bed reactor with the immobilized racemase showed good performance in steady state operation processing low enantiomeric excess inlet. Kinetic parameters from batch reactor experiments can be successfully used for prediction of packed bed reactor performance. Full conversions could be achieved for residence times above 1.1 min. The results suggest the potential of the prepared racemase reactor to be combined with preferential crystallization to improve resolution of asparagine enantiomers.

**Abbreviations**
AAR: amino acid racemase
PBR: packed bed reactor
PC: preferential crystallization
PLP: pyridoxal 5′‐phosphate monohydrate

## 1 INTRODUCTION

Manufacturing optically pure compounds is important for the pharmaceutical and fine chemical industries. A pair of enantiomers have identical properties, with exception of interactions with polarized light and with biomolecules, often leading to different effects in living organisms. For that reason, in the past few decades many blockbuster drugs experienced the chiral‐switch from pre‐marketed racemates, and the strong majority of the chiral drugs recently approved by the Food and Drug Administration are single enantiomers [1]. The method of choice to obtain a pure enantiomer largely depends on the specific target compound. Most production routes are based on asymmetric synthesis, using stereoselective catalysts, or on producing racemic mixture followed by a resolution process [2]. This last approach is increasingly economically attractive. However, since in most cases only one enantiomer is the target product, the overall separation suffers from a maximum theoretical yield of 50% with respect to the total starting material. Racemization of the undesired enantiomer is attractive as it allows overcoming this limitation. In addition to improving the process yield, a coupling of resolution and racemization promotes recycling of the non‐target compound and reduces waste. The use of enzymes has an increasing impact on industrial processes [3] and it is an attractive alternative to chemical catalysis due to biodegradability, high selectivity, and the use of mild reaction conditions regarding solvent, temperature and pressure [4].

**FIGURE 1:**
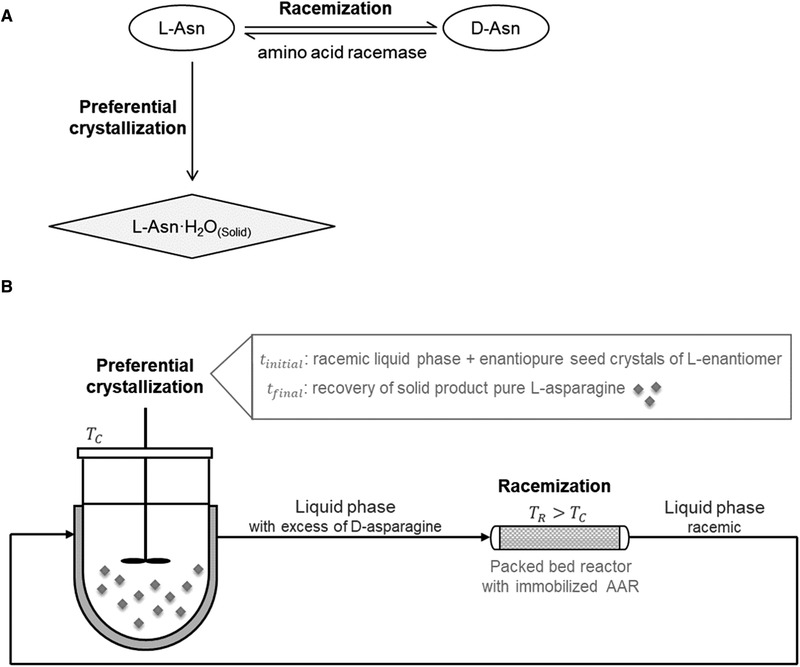
(A) Schematic process and (B) setup for a direct coupling of preferential crystallization and packed bed racemization reactor for production of L‐asparagine. The feed is racemic. The target enantiomer is removed from solution via crystallization. The excess of D created in solution is racemized in the enzymatic reactor. No solid phase should be withdrawn from the crystallizer. The reactor must be operated at a higher temperature than the crystallizer ($T_{R}$ > $T_{C}$) to avoid crystallization outside the appropriate unit

The coupling of a resolution process with racemization has been demonstrated using, for instance, dynamic kinetic resolution [5], chromatography [6, 7, 8], preferential crystallization [9] or a combination of the last two techniques [10, 11]. Crystallization‐based methods have the advantage of being less expensive than chromatography and they result in a solid product, which is often the desired form in the industry. Preferential crystallization (PC) allows the direct resolution from a racemic feed for conglomerate‐forming systems [2]. Conglomerates crystallize as a mechanical mixture of the enantiomers, with no heterochiral solid phase. PC has been investigated for separation of enantiomers in various modes, e.g. batch or continuous, stirred tanks, fluidized beds, and in combination with chromatography [2, 12]. The resolution starts by seeding a supersaturated racemic mixture with pure chiral crystals to induce crystallization of the target enantiomer. In this work we focus on the combination of batch PC and racemization (Figure 1A). Asparagine monohydrate was considered as a model substance because it crystallizes as a conglomerate, and it can therefore be separated by PC starting from the racemic mixture [13]. Deracemization processes based on grinding or temperature cycling are alternative crystallization techniques, which require racemization in the liquid phase to produce enantiopure compounds [14, 15, 16]. All the above described crystallization processes have in common the generation of rather low enantiomeric excesses, therefore, low driving force for the racemization reaction. To compensate for this fact higher dosages of catalyst must be employed.

**PRACTICAL APPLICATION**

Production of single enantiomers is essential for studying and commercializing pharmaceuticals. Pure chiral molecules can be obtained by resolution of racemic mixtures, but these separation methods suffer from a maximum yield of 50% with respect to the desired enantiomer. In this work, we investigated the recycling of the undesired enantiomer into the racemic substrate by a racemase. We propose the combination of preferential crystallization, a cost‐effective resolution process, with a packed bed reactor containing the immobilized racemase. This process design has the potential to maximize yield to 100% with high product purity, while providing convenient enzyme‐product separation and catalyst reuse. This study shows the development of the enzymatic reactor and provides a starting tool for estimating parameters and operating conditions for the process combination. It shows the great potential of the enzymatic reactor to improve the chiral resolution.

The enzyme chosen in our work is a well‐known amino acid racemase (AAR) (EC 5.1.1.10) from Pseudomonas putida KT2440 cloned into *Escherichia coli* (*E. coli*). The racemase is pyridoxal 5′‐phosphate monohydrate‐dependent (PLP‐dependent), it has a broad substrate specificity and potential for preparative biotransformations [17]. Kinetic properties of different preparations of this enzyme have been reported with several substrates [18, 19, 20]. A previous study has shown a proof of concept of the combination of PC and in situ racemization with the AAR in a 20 mL reactor [9]. However, catalyst‐product separation is an issue in one‐pot combinations. Additionally, the presence of the soluble enzyme plays an unknown role on the solubility of the crystallizing molecule.

For a cost‐efficient process, recycling of the catalyst is essential. Enzyme immobilization provides recyclability, stabilization, and easy separation from reaction media [21, 22]. Immobilized biocatalysts also enable the application of packed bed reactors (PBR). PBRs have been employed for the combination of enzymatic racemization with chromatography [8, 23], but never before with preferential crystallization. Covalent immobilization is an enzyme‐binding approach that has the advantage of building a strong, often multipoint attachment, which prevents leaching of the catalyst [4]. A down‐side of such robust binding is that it can chemically modify the enzyme and may decrease its activity. In addition, it requires a relatively demanding processing time, that may include pre‐activation step and/or blockage of uncoupled binding sites [24].

We propose for the first time the combination of PBR to improve enantioresolution with preferential crystallization. Figure 1 shows a schematic process and setup of the desired combination. In our previous work, we developed a model to quickly assess productivity of preferential crystallization and reported PC experiments with asparagine monohydrate [25]. The goal of the present study is to provide an effective bioreactor that can be combined with preferential crystallization of asparagine enantiomers. The approach presented here comprises the improvement of the purification of the racemase, as well as finding an optimal immobilization strategy to obtain a stable and easily reusable catalyst. A Histidine‐tag was added to the enzyme to facilitate its purification. Racemization kinetics of pure free racemase was determined at different temperatures and the AAR behavior was evaluated for various reaction conditions such as dosage (relative amount of enzyme) and initial enantiomeric excess. Immobilization of the AAR was tested on three covalent binding supports. Kinetic parameters were determined for the most promising immobilized AAR. Finally, we reported results on the racemase performance in a packed bed reactor at steady state conditions.

## 2 MATERIALS AND METHODS

### 2.1 Materials

Isopropyl‐β‐D‐thiogalactopyranosid (IPTG) was obtained from Carl Roth GmbH and Co. KG. Protease inhibitor cocktail set VII from Calbiochem was purchased from Merck KGaA and PLP from Sigma‐Aldrich. Immobilization supports Lifetech™ ECR were kindly donated by SpinChem AB and Purolite^®^. Enzyme carrier Eupergit^®^ CM was purchased from Sigma‐Aldrich. D‐asparagine monohydrate was purchased from Alfa Aesar and L‐asparagine monohydrate from Sigma Aldrich chemicals, both of purity ≥99%.

### 2.2 Construction of His‐tagged amino acid racemase

To facilitate purification of amino acid racemase, a C‐terminal His‐tag was added to AArac12996 from *P. putida* KT2440 encoded on plasmid pET‐22b [26]. For insertion of additional bases encoding a C‐terminal His‐tag, a PCR‐based strategy was applied, using the Q5 Site‐Directed Mutagenesis Kit (New England Biolabs) and primers AAR‐mut‐for (gtcgacgagtatcttcgggtt) and AAR‐mut‐rev (gagcaccaccaccaccacca). The sequence of the mutated plasmid was verified by sequencing.

### 2.3 Enzyme production and purification

The recombinant His‐tagged AAR was overexpressed in *E. coli* BL21 (DE3). The cells were grown in 12 L LB medium enriched with 0.1 g/L ampicillin, 10 g/L glucose, 2.5 mM CaCl2 at 30°C. To induce the expression of the AAR, 0.1 mM IPTG was added at optical density OD_650nm_ ≈ 1.0. The temperature was set to 22°C and the cells grew until OD_650nm_ ≈ 2.8. Total fermentation time was about 7 h. Cell suspension was fractioned, harvested by centrifugation (5000 g, 50 min, 4°C) and stored at −20°C. The overexpression produced 4.5 g of wet pellet/L fermentation.

Protein purification was performed by affinity chromatography using HisTrap FF crude 5 mL column (GE Healthcare) carried out in an Äkta system (Purifier 25, GE Healthcare) at 4°C. Cell pellet was resuspended in lysis buffer (20 mM sodium phosphate buffer with 500 mM NaCl, pH 7.4) with 0.1 mL/g‐wet pellet of protease inhibitor. The cells were disrupted either by sonication (Ultrasonic cell disruptor 450d, Branson; amplitude 65%, 3 min, cycles of 0.5 s and 12 s break) followed by centrifugation at 17 000 g (4°C, 30 min), or by high‐pressure homogenization (EmulsiFlex‐C5, Avestin Inc.) over maximum pressure drop of 2500 psi, followed by centrifugation at 25 000 g (4°C, 20 min). Cofactor PLP was added to achieve concentration of 50 μM. After loading the crude extract (CE) solution, non‐specific binding was washed out and the AAR was recovered by stepwise gradient elution: 2 CV from 0 to 65% elution buffer, followed by 2 CV at 65% and 2 CV from 65 to 100%. The washing and elution buffers had a similar composition and pH of the lysis buffer with additional 15 and 300 mM imidazole, respectively. The purified fractions were evaluated by SDS‐PAGE analysis.

### 2.4 Enzyme immobilization

Prior to immobilization, the purified AAR solution was concentrated using Vivaspin 15 (Sartorious AG) and the buffer was exchanged to a suitable immobilization buffer using Zeba™ Spin desalting columns of 5 mL with 7K MWCO (Thermo Fischer Scientific Inc.). Although necessary, this step resulted in average loss of 18% of enzyme mass and of 20% of specific activity.

#### 2.4.1 AAR immobilization on Eupergit^®^ CM

Purified AAR solution in 1.0 M potassium phosphate buffer pH 7.4 was added to dry support Eupergit CM (particle size 50–300 μm, pore size 500 Å) for protein load of 27 and 37 mg‐enzyme/g‐support. The initial enzyme concentration was 1 mg/mL. The slurry was incubated at room temperature for 72 h in a rotating mixer. The support with immobilized enzyme was filtrated and washed with 10 mM potassium phosphate buffer pH 7.4. The uncoupled binding sites were blocked in a solution of 50 mM glycine for 1 h.

#### 2.4.2 AAR immobilization on Purolite Lifetech™ ECR 8204

The carrier ECR 8204 (particle size 150–300 μm, pore size 300–600 Å) was equilibrated by washing with lysis buffer. Next, 0.3 g of support incubated with purified AAR in 0.5 M sodium phosphate buffer at pH 7.4 at protein load of 25 and 35 mg‐enzyme/g‐support. A resin/buffer ratio of 1/4 w/v was used. The slurry was put in a rotating mixer for 18 h and was subsequently left without mixing for 20 h at room temperature. The AAR support was then filtrated and washed with lysis buffer.

#### 2.4.3 AAR immobilization on Purolite Lifetech™ ECR 8309

The support ECR 8309 (pore size 600–1200 Å) was first equilibrated by washing with lysis buffer and activated for 1.5 h with 2% glutaraldehyde. Purified AAR solution was added to typically 0.25–0.30 g of activated support in a resin/buffer ratio of 1/4 w/v and kept for 18 h at room temperature. The following incubation loads were tested: 4.2, 25, 35, and 50 mg‐enzyme/g‐support for ECR 8309F (particle size 150–300 μm) and 4.2  mg‐enzyme/g‐support for ECR 8309 M (particle size 300–710 μm). The immobilized support was filtrated and washed with lysis buffer.

### 2.5 Protein quantification

Bradford assay [27] was used to measure protein concentration in solution with bovine serum albumin as standard. The amount of protein adsorbed ($m_{\mathrm{adso}\mathrm{rbed}}$) was determined by the difference between the protein in solution before and after immobilization, including filtrate solution and subsequent washing steps. The immobilization achieved was evaluated in terms of specific activity and immobilization yield ($Y_{\mathrm{im}}$). The yield was calculated from the ratio between the mass of protein adsorbed and the initial mass (*m* ^0^) before immobilization (Equation 1). Immobilization was also evaluated qualitatively with SDS‐PAGE analysis.

(1) $$Y_{\mathrm{im}}\left[\%\right]=\frac{m_{\mathrm{adso}\mathrm{rbed}}}{m^{0}}\times 100$$

### 2.6 Enzyme activity

#### 2.6.1 Activity assay for free enzyme

##### Polarimetric method

Investigated protein solution was added to achieve a defined catalyst dosage $D_{C}$ (see below Equation 4) to 3 g solution of asparagine in water, which was rapidly injected into a polarimetric cell (MCP 500 Modular Circular Polarimeter, Anton Paar). The process of racemization was followed by monitoring the decrease in optical rotation at a wavelength of 365 nm.

Kinetic parameters of purified enzyme were determined at 30, 35, and 40°C in a range of initial substrate concentrations between 0.11 and 5.0 wt% D‐Asn monohydrate (7.6–333 mM). The catalyst dosage was 30 mg‐enzyme/L. All measurements were repeated twice.

The influence of enantiomeric excess (5, 10, 20, and 100%) in specific activity was tested for substrate solutions with D‐Asn·H_2_O concentrations of 3.2, 4.4, and 5.3 wt% (213, 293, and 353 mM) for reactions at 30, 35, and 40°C, respectively. The respective enantiomeric excesses were created by adding L‐Asn·H_2_O in different amounts (Equation 7). In these runs, the catalyst dosage was 20 mg‐enzyme/L.

##### HPLC method

Samples with higher enzyme concentrations could not be evaluated by the Polarimetric Method because the presence of the protein resulted in a deviation of optical rotation from linear behavior. HPLC analysis was used to study the influence of catalyst dosage in the racemization for $D_{C}$ = 20, 100, and 400 mg‐enzyme/L. The reaction was carried out in 1 mL of 2.5 wt% D‐Asn·H_2_O (167 mM) in Eppendorf Thermomixer (Eppendorf AG) at 1000 rpm and 30°C for 1 to 15 min. The reaction was terminated by diluting the reaction media in perchloric acid pH 1.0.

##### HPLC analysis

The reaction compositions were analyzed using a Dionex UltiMate 3000 HPLC (Thermo Fischer Scientific Inc.) equipped with Crownpak CR (+) (length 150 mm, inner diameter 4.0 mm; Daicel corporation, Chiral Technologies Europe SAS). The separation was performed at 5°C with perchloric acid pH 1.0 as mobile phase, with flow rate 0.4 mL/min and 1 μL injections and UV/Vis detection at 200 nm. The retention times were 2.9 and 3.5 min for d‐ and L‐asparagine, respectively.

#### 2.6.2 Activity assay for immobilized enzyme

Immobilized biocatalyst (20 mg of Purolite ECR carriers or 40 mg of Eupergit CM) was added to 1 mL of D‐Asn·H_2_O solution 2.5 wt% (167 mM). The suspension was agitated at 1000 rpm and 30°C in an Eppendorf Thermomixer for 2 to 8 min. The reaction was terminated by filtration of the reaction media with Rotilabo PET‐membrane syringe filters (Carl Roth GmbH+Co. KG). All measurements were repeated twice. The solution compositions were analyzed with HPLC using the same protocol described above.

##### Quantification of enzyme kinetics

The racemization kinetics of an amino acid racemase can be described by a reversible three‐step Michaelis‐Menten mechanism [17]. Assuming the reaction kinetics is equal for both enantiomers, the following equation is given:

(2) $$r=V_{\max}\;\frac{c_{D}-c_{L}}{K_{M}+c_{D}+c_{L}}$$

where $V_{\max}$ and *K*_M_ are the kinetic parameters, *c*_D_ and *c*_L_ are the concentration of enantiomers D and L. In a batch reactor, the mass balance becomes:

(3) $$\frac{dc_{i}}{dt}=\nu_{i}\;D_{C}\;r\;i=D,L$$

where ν_i_ is the stoichiometric coefficient of the enantiomer i, which corresponds to −1 or 1 when d‒amino acid is the reaction substrate or product, respectively. The catalyst dosage *D*_C_ (Equation 4) is the concentration of the biocatalyst in the reaction volume *V*_R_, and it is expressed in mg‐enzyme/L for free purified enzyme, or in g‐support/L in case of immobilized AAR.

(4) $$D_{C}=\frac{m_{\mathrm{catalyst}}}{V_{R}}$$

The enzyme *specific activity* can be calculated from the reaction rate of racemization of pure D enantiomer in the beginning of the reaction:

(5) $$r_{0}=r\left(t\to 0\right)=V_{\max}\;\frac{c_{D}}{K_{M}+c_{D}}$$

The specific activity is expressed in units U per milligram of free enzyme or per gram of immobilized support, where 1U corresponds to 1 μmol of substrate converted per minute of reaction. In the case of substrate inhibition, another kinetic parameter *K*_I_ must be considered to calculate the specific activity:

(6) $$r_{0}=r\left(t\to 0\right)=V_{\max}\;\frac{c_{D}}{K_{M}+c_{D}+\frac{c_{D}^{2}}{K_{I}}}$$

### 2.7 Kinetics of racemization in a batch reactor

AAR immobilized in ECR 8309F at protein load of 35 mg‐enzyme/g‐support was used in kinetic measurements. A total of 0.4 g of wet support was added to 50 mL asparagine solution in a double jacket vessel equipped with overhead magnetic stirrer (Wheaton, DWK Life Sciences Inc.). Online monitoring of the reaction process was obtained by pumping solution at 8 mL/min through a polarimeter. Sintered glass filters were used to prevent removal of immobilized enzyme from the reactor. The reactive solutions were held at 40°C with stirring speed of 300 rpm and initial D‐Asn·H_2_O concentration ranging from 0.11 to 5.0 wt% (7.6 to 333 mM). Each measurement was repeated twice.

### 2.8 Steady‐state racemization in packed bed reactor

**FIGURE 2:**
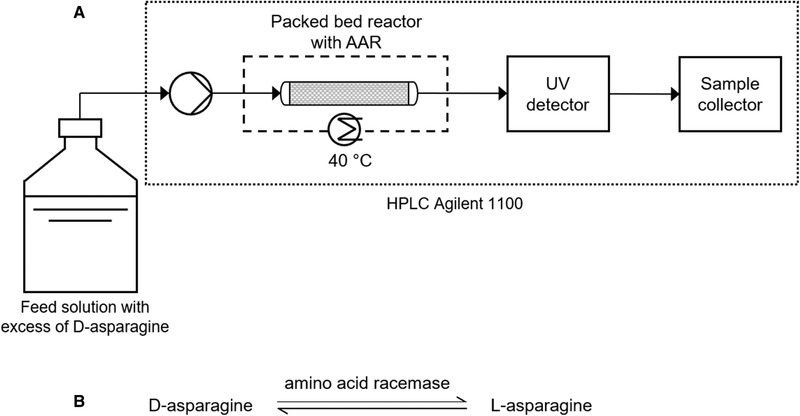
(A) Schematic experimental setup: enzymatic packed bed reactor with immobilized AAR for racemization of asparagine in water. The feed solutions were pure D‐Asn (ee_0_ = 100%) and D‐/L‐Asn with excess of D (ee_0_ = 4.5%). (B) Reaction sequence

In final experiments, the AAR purified and immobilized on enzyme support ECR 8309F with load of 35 mg‐enzyme/g‐support was used. It was packed in a Tricorn column 5/100 (GE Healthcare). The resulting bed height and catalyst mass were 10.8 cm and 1.84 g, respectively. The reactor volume was 2.12 mL. The packed bed reactor (PBR) was connected to an HPLC system (Agilent Technologies 1100) equipped with a column oven set at 40°C. A representation of the experimental setup and the reaction are shown in Figure 2. The inlet substrate solutions were asparagine monohydrate in water 2.5 wt% with initial enantiomeric excess of D‐Asn·H_2_O of 4.5% and 100%. The reactions were performed by constantly pumping substrate solution over the PBR at flow rates in the range 0.1–3.5 mL/min until steady state was reached. This equilibrium was verified via the formation of a plateau in the signal of UV detector. Then, samples of the outlet flow were collected and analyzed using the *HPLC analysis* protocol described in Section 2.6.1.

## 3 RESULTS

### 3.1 Kinetics of free enzyme

Kinetics of pure free amino acid racemase was tested at 30, 35, and 40°C (Figure 1A). The three kinetic profiles reached a maximum before dropping, suggesting an inhibition effect. This kinetic behavior was well described by the enzymatic activity accounting for substrate inhibition (Equation 6). The kinetic parameters $V_{\max}$, *K*_M_, and *K*_I_ were estimated for each temperature (Table 1). The overall specific activity increases at higher temperatures, which is reflected by the increase of $V_{\max}$. Values of *K*_M_ declined with increasing *T*, indicating that at higher temperatures there is a higher frequency of formation of enzyme‐substrate complex. The parameter *K*_I_ increased with *T* and the same trend was observed for the ratio $K_{I}/K_{M}$, suggesting a lower influence of this inhibition at higher temperatures [28].

**TABLE 1:** Kinetic parameters estimated for purified free and immobilized AAR^a^ ^)^

AAR	T [°C]	V_max_ [U/mg‐enzyme]	V_max_ [U/g‐support]	K_M_ [mM]	K_I_ [mM]
Free	30	22 ± 4.3		42 ± 15	238 ± 101
Free	35	24 ± 2.0		31 ± 5.0	496 ± 129
Free	40	30 ± 2.1		20 ± 3.3	794 ± 234
Immobilized in ECR 8309F	40	37 ± 4.3^b)^	1309 ± 122	201 ± 36	

^a)^ Estimation of parameters by nonlinear regression using OriginLab 2019.

^b)^ The parameter $V_{\max}$ for the immobilized racemase was calculated in mg‐enzyme from the respective immobilization load (35 mg/g‐support).

**FIGURE 3:**
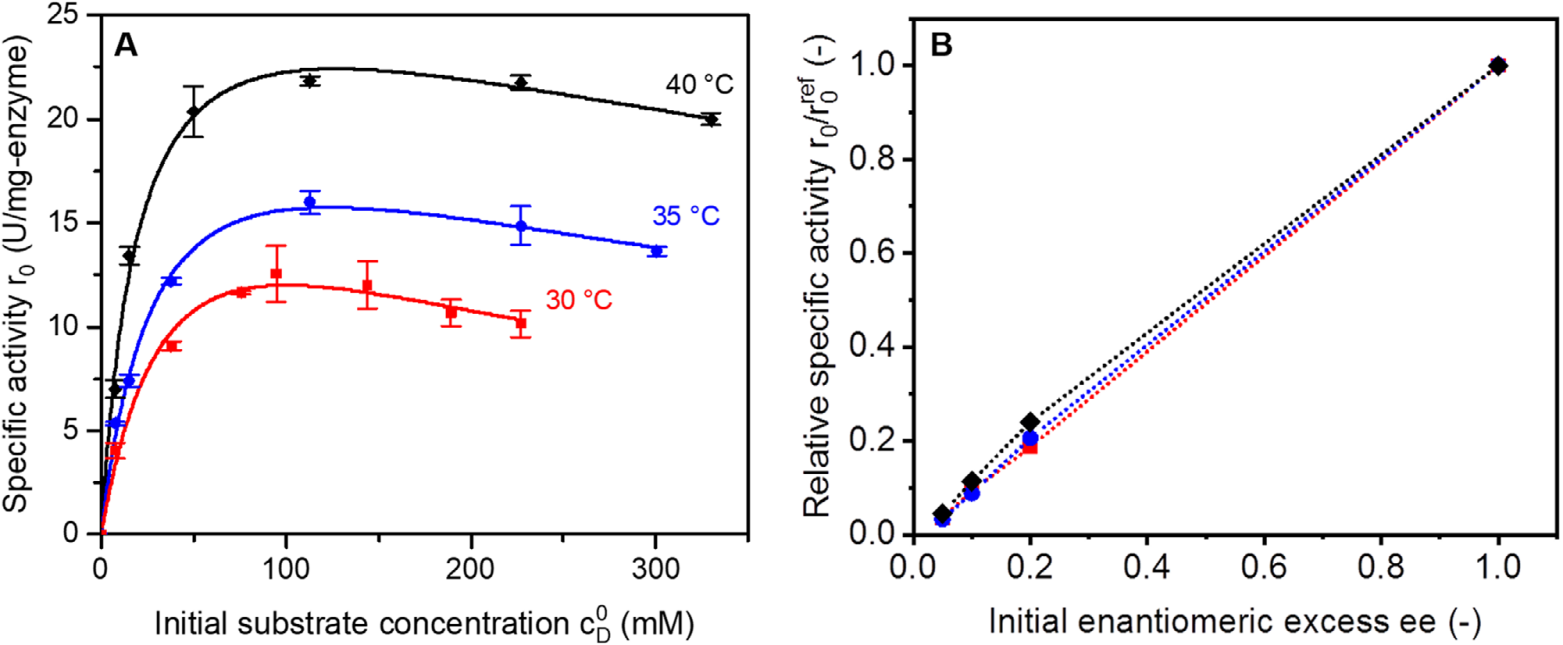
Specific activity of purified free enzyme at 30 (red squares), 35 (blue circles), and 40°C (black diamonds). (A) Racemase kinetics. Solid curves are the kinetic functions (Equation 6) using the estimated parameters at each temperature. Reaction substrate: pure D‐Asn in water; D_C_ = 30 mg‐enzyme/L. The experiments were performed in duplicate. (B) Effect of initial enantiomeric excess on activity of free AAR. Specific activity was calculated relative to the maximum found activity (when ee^0^ = 1.0) at each respective temperature. Experimental conditions: D‐Asn·H_2_O in 3.2, 4.4 and 5.3 wt% at 30, 35 and 40°C, respectively; initial enantiomeric excesses 0.05, 0.1, 0.2 and 1.0 created by addition of necessary amount L‐Asn·H_2_O (Equation 7); D_C_ = 20 mg‐enzyme/L

The influence of the amount of enzyme on the reaction rate was evaluated for dosages 20, 100, and 400 mg‐enzyme/L. Although uncommon, for some enzymes at certain reaction conditions a nonlinear effect between activity and dosage can be observed [29]. Therefore, we confirmed experimentally the influence of catalytic dosage of AAR. As expected, there was a proportional relation between amount of enzyme and rate of reaction. By increasing the dosage 5 and 20‐fold, the initial rate increased linearly in the same way. This is an important finding since high amounts of enzyme are needed to compensate for low driving forces expected in the desired coupling (Figure 1B). Preferential crystallization breaks the racemic solution symmetry by crystallizing one of the enantiomers selectively and can successfully be used as a separation method. However, it tends to generate only low enantiomeric excess ee (Equation 7) in the liquid phase. To efficiently improve the enantioresolution, racemization must take place at conditions near to its equilibrium. The effect of initial enantiomeric excess on the specific activity was investigated at three temperatures at concentration values close to the respective solubility limits. The results are shown in Figure 3B. For comparison, the values of specific activity were calculated relative to the reference activity at each temperature, when ee = 100%. From the results, it is obvious that the initial ee determines the reaction driving force and has a strong impact on its rate.

(7) $$\mathrm{ee}=\frac{c_{D}-c_{L}}{c_{D}+c_{L}},\;\mathrm{ee}\left[\%\right]=100\;\mathrm{ee}$$

### 3.2 AAR immobilization

Three supports were tested for immobilization of purified AAR: Eupergit^®^ CM, and Purolite Lifetech™ ECR 8204F and ECR 8309 (in particle size of grades F and M). Both Eupergit CM and Lifetech ECR 8204F are microporous, epoxy‐activated acrylic supports. The immobilization process occurs via multipoint covalent binding between the epoxy groups of the acrylic polymer and functional groups of the protein. Lifetech ECR 8309 is an amino‐activated support with ethylene spacer and it must be pre‐activated with glutaraldehyde. The immobilization reaction occurs between the aldehyde and amino groups of the enzyme to form a Schiff base.

**FIGURE 4:**
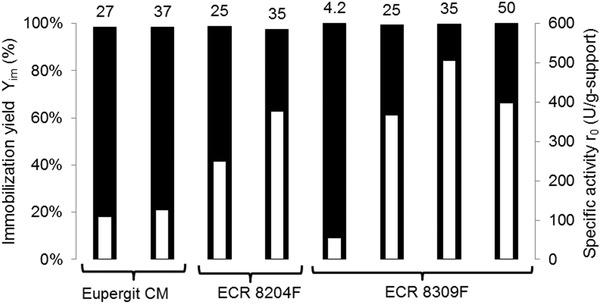
Investigation of immobilization of purified AAR for distinct incubation loads (4.2–50 mg‐enzyme/g‐support, indicated above the bars). Black bars: immobilization yield (Equation 1); white bars: specific activity

Initially, Eupergit CM was tested at incubation loads 27 and 37 mg‐enzyme/g‐support and the Purolite carriers at incubation loads 25 and 35 mg‐enzyme/g‐support. The results are presented in Figure 4. All covalent‐binding supports achieved immobilization yields higher than 97%. In the above‐mentioned load range, all three supports presented a higher specific activity for the highest load. The highest activity was obtained for ECR 8309F. A possible effect of pore size could not be quantified based on our results. Finally, the influence of the incubation load for this resin was tested for a broader range of 4.2 to 50 mg‐enzyme/g‐support (Figure 4). The amount of bound protein was high for the entire range of initial load, with immobilization yields above 99%. When the load was increased from 35 to 50 mg‐enzyme/g‐support, the specific activity decreased. This result is probably a consequence of steric hindrance caused by high density of immobilized enzyme. This may limit the accessibility to the active site or in some cases even cause protein denaturation [30]. The highest specific activity of 506 U/g‐support was obtained for an incubation load of 35 mg‐enzyme/g‐support. This carrier and immobilization conditions were chosen for further experiments.

The carrier with a larger particle size, namely ECR 8309 M, was tested at incubation load of 4.2 mg‐enzyme/g‐support. At similar conditions, 22% higher activity was obtained with grade F material. This result is possibly a consequence of mass transfer limitations or hindered accessibility to the active site in the larger support [4, 30]. However, further experiments would be needed to fully clarify the results. Additionally, immobilization of the AAR crude extract on ECR 8309F was tested. Before purification the extract presented rather low specific activity (0.5 to 1.0 U/mg‐protein), which was improved upon immobilization. Nevertheless, for a similar incubation load the activity of the immobilized purified racemase was 94% higher than that of the immobilized crude extract.

The recyclability of the immobilized AAR was tested by incubating for 30 min, 40 mg of carrier with 1 mL substrate for six cycles. The final concentration was racemic in all cycles and no loss in activity was observed. The good stability of the immobilization binding was confirmed during the experiments in packed bed reactor, as it is shown in the following.

### 3.3 Kinetics of immobilized enzyme in a batch reactor

**FIGURE 5:**
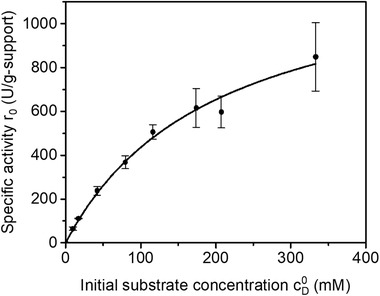
Kinetics of AAR immobilized on ECR 8309F with load 35 mg/g‐carrier. Symbols: experimental points; solid curve: Michaelis‐Menten equation (Equation 5) with estimated parameters (Table 1). Reaction substrate: pure D‐Asn in water; D_C_ = 8.0 g‐support/L, T = 40°C. The measurements were taken in duplicate

The specific activity of the AAR immobilized on ECR 8309F was studied for various initial concentrations of D‐Asn. The kinetic parameters of the Michaelis‐Menten equation were determined and the results are shown in Figure 5 and Table 1. Unlike the results for free enzyme, no effect of inhibition was detected in the kinetics of the immobilized AAR. The reduction or hindrance of inhibition effects upon immobilization has been previously reported [22, 31]. As a consequence, $V_{\max}$ of the immobilized AAR was higher than the value obtained for the free enzyme. The value of *K*_M_ was almost 10 times higher than that of the free enzyme at the same temperature. This reflects an apparent lower affinity to the substrate, which is probably a result of mass transfer effects during the reactions with immobilized enzyme [30].

### 3.4 Performance of immobilized AAR in packed bed reactor

To reach the initially expressed goal, that is, to investigate the behavior of the racemase for the combination with preferential crystallization, a column was packed with immobilized AAR. The mass balance for a packed bed reactor (PBR) operated at steady state conditions can be written as [32]:

(8) $$\frac{dc_{i}}{d\tau }=\nu_{i}\;D_{C}\;r\;i=D,L$$

which is analogous to the mass balance of a batch reactor described in Equation 3, but dependent on the residence time τ instead of the real time t. Hereby τ is defined as:

(9) $$\tau =\frac{V_{R}}{\dot{V}}$$

where *V*_R_ is the reaction volume, correspondent to the volume of the bed, and $\dot{V}$ is the volumetric flow rate.

In any direct process coupling, similar operating conditions in terms of, e.g. solvent are highly advantageous. The combination of preferential crystallization and packed bed racemization reactor is foreseen in a simultaneous online process (Figure 1) or in a stepwise manner. In both cases, racemization should be held at a higher temperature than the resolution process. Since PC is performed in a supersaturated state, having different operating temperatures in each unit would prevent crystallization in the catalytic reactor. Racemization at 40°C provided the highest activity for free enzyme among the range tested (Figure 3A) and therefore this condition was chosen for racemization in packed bed reactor. Coupled PC must then be implemented at a lower saturation temperature. In addition, as shown in Section 3.2, the enzymatic activity drops significantly when the initial enantiomeric excess is low. This is an essential parameter when combining preferential crystallization with racemization. We measured a maximum enantiomeric excess of 4.6 to 5.1% reached during isothermal preferential crystallization of asparagine monohydrate at 30°C with saturation at 35, 37, and 40°C [25]. To evaluate the effect of ee on the conversion in PBR, the packed column was fed with asparagine solutions with ee equal to 4.5% and 100% (excess of D‐Asn). Figure 2 gives an illustration of the experimental setup used.

Several residence times were tested for each solution. The concentrations at column outlet were analyzed at steady state conditions. For each residence time the conversion was calculated as a function of the initial enantiomeric excess ee^0^:

(10) $$X_{\mathrm{ee}}=\frac{ee^{0}-\mathrm{ee}}{ee^{0}}=\frac{\left(c_{D}^{0}-c_{D}\right)-\left(c_{L}^{0}-c_{L}\right)}{c_{D}^{0}-c_{L}^{0}}$$

If instead the conversion would have been calculated using the conventional expression as a function of the substrate concentration, for example ($X=\left(c_{D}^{0}-c_{D}\right)\;/c_{D}^{0}$), the equilibrium conversion *X* ^eq^ would be dependent on the initial enantiomeric excess of the substrate. For instance, for ee^0^ = 100%, *X* ^eq^ = 0.50 and for ee^0^ = 4.5%, *X* ^eq^ = 0.043. Using Equation 10, the maximum equilibrium conversion is 1.0 for all ee^0^ conditions.

**FIGURE 6:**
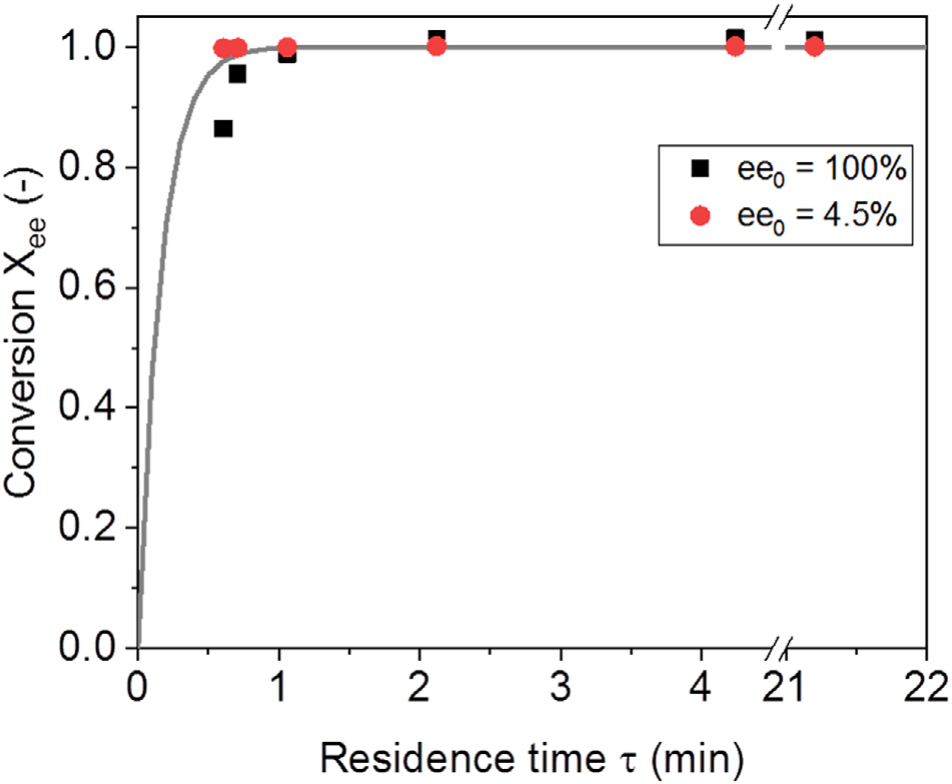
Influence of residence time τ and enantiomeric excess ee on the racemization in a packed bed reactor. Each data was analyzed at steady state. Red circles are ee^0^ = 4.5% and black squares are ee^0^ = 100%, both in excess of D‐Asn. Solid curve: simulation of PBR using kinetic data for the immobilized racemase. Reaction conditions: AAR immobilized in ECR 8309F, load 35 mg/g‐carrier; dosage 868 g‐carrier/L; 40°C; substrate concentration: 2.5 wt% (167 mM) asparagine monohydrate in water

The results of packed bed reactor at steady state are presented in Figure 6. For the substrate solution with ee^0^ = 4.5%, conversions above 0.998 were obtained for all values of residence time tested. When starting the reaction with pure enantiomer (i.e. ee = 100%), the racemization achieved a minimum conversion of 0.865 at τ = 0.6 min. After 1.1 min, conversions higher than 0.990 were reached. All reactions performed at residence times longer than 2 min generated slightly negative values of ee% (−0.4 to −1.5). Since enzymatic racemization does not proceed beyond the racemic composition, it can be assumed that the negative values of ee were rather related to sensitivity in the analytics towards very low enantiomeric excesses.

Biocatalytic stability was verified by repeating the experimental condition of initial ee = 100% at the lowest τ after two full days of experiments and five days of storage at 4°C. The measured conversion resulted in 93% of the initial, which proves stability of the packed column.

The kinetics of the immobilized amino acid racemase obtained in stirred batch reactor (parameters showed in Table 1) was used for modelling the packed bed reactor (Equation 8). The experimental profiles were consistent with simulations (Figure 6). The determination of a more detailed conversion profile, with experiments in the range below τ = 1.1 min, was out of the scope of this study. In addition, measurements below the lowest residence time used (τ = 0.6 min, or flow rate 3.5 mL/min) were not possible to execute due do limits in back pressure of the system.

## 4 DISCUSSION

His‐tagged amino acid racemase was produced and purified with help of affinity chromatography. Influence of substrate inhibition was found for the free enzyme kinetics at 30, 35, and 40°C (Figure 3A). The reaction velocity increased with increasing temperature, and it caused the inhibition effect to weaken. In a previously reported work [9], the same AAR was purified via fractionated precipitation with subsequent ion exchange chromatography. The authors reported a typical Michaelis‐Menten kinetics, with no effect of substrate inhibition observed. Nevertheless, with the production and purification protocols proposed in the present work there was a substantial improvement in specific enzymatic activity, with higher values of $V_{\max}$ (7 to 12‐fold, depending on temperature) and lower values of *K*_M_ (1.5 to 5.1‐fold). Therefore, the enzyme preparation procedure presented in this paper can be considered more efficient than the protocol previously proposed.

A strong impact of amount of enzyme (catalyst dosage *D*_C_) and initial enantiomeric excess on the enzymatic racemization activity was observed. In particular, the initial ee is an important parameter for integration of preferential crystallization and enzymatic reaction. The operating window for the coupling with PC is placed closer to the lower left corner of Figure 3B. For this reason, high dosages of enzyme have to be used so that the racemization is not the rate limiting step of the coupling and effectively improves the chiral resolution. Such limitation is not an issue for integration with other types of enantioresolution. A similar amino acid racemase in a membrane reactor was applied in combination with SMB chromatography to improve separation of methionine enantiomers. This method generates reaction substrates highly enriched in unwanted enantiomer and therefore low amounts of enzyme are need [7]. The main limitation regarding the biocatalyst was stability in organic solvent mixtures, which has been recently showed to significantly improve by enzyme engineering [33]. In dynamic kinetic resolution, the racemization must be at least as fast as the enantioselective reaction for the coupling to be effective [34]. A N‐succinyl‐amino acid racemase was used in a bienzymatic dynamic kinetic resolution system for production of several pure amino acids. The racemase immobilization loading and enzyme dosage were tailored to compensate for reaction rates lower than that of the enantioselective enzyme [35].

Enzyme immobilization brings the advantage of easy separation from reaction media and re‐use, in addition to the possibility of using a high concentration of enzyme for the proposed application in combination with PC (Figure 1). Since enzyme production is expensive and time consuming, recyclability of the catalyst is crucial. High enzymatic activity and effective recyclability was obtained over numerous cycles for immobilization on ECR 8309F at load of 35 mg‐enzyme/g‐support. Substrate inhibition was not observed upon immobilization (Figure 5), which is important since preferential crystallization takes place at high substrate concentrations, close to solubility limits.

The efficiency of the enzymatic flow reactor with immobilized AAR was validated in steady state experiments at two different enantiomeric excesses, 100 and 4.5%. This last condition is expected during PC of asparagine enantiomers, according to our previous experiments [25]. A minimum residence time of only 1.1 min was needed to fully convert the substrate into the racemic mixture regardless of the initial ee. Under the conditions tested, the reactor performance was successfully predicted using kinetic parameters estimated from results of batch runs. Even though the PBR profile would profit from characterization below 0.6 min residence time (Figure 6), the kinetic information reported here allows for simulations of the reactor behavior. That, together with key data from preferential crystallization form a good quantification basis for overall process design, which can be used as a prediction tool for estimation of the performance of this promising coupling over a range of process conditions (e.g. enzyme dosage and volumetric flow rate).

The high stability and high activity of the racemization reactor make it an attractive complementary unit operation for preferential crystallization. Especially the capability of rapid complete racemization even at low enantiomeric excesses is crucial for the integration with crystallization‐based resolution methods that generate low driving forces for racemization. This is also valid for deracemization techniques via grinding and temperature gradient, which have been mostly reported with in situ chemo‐catalytic racemization [36, 37]. The results described in this paper can be useful to apply the concept of combining preferential crystallization and racemization in enzymatic flow reactor to improve the provision of enantiomers of numerous other chiral organic molecules. Additional potential is seen in its use with other enantioselective resolution techniques, such as chromatography.

Finally, it should be stated that in this work we focused on the production of L‐Asn, with D‐Asn as the substrate of the enzymatic reactions. Nevertheless, both the racemase and the preferential crystallization process can be applied for the conversion and crystallization of D‐ or L‐Asn. The same process concept can be used for the production of this and other pure D‐amino acids, which are often significantly more expensive than L‐amino acids.

## CONFLICT OF INTEREST

The authors have declared no conflict of interest.
